# Cervical cancer prevention in Riyadh: a cross-sectional analysis of sociodemographic correlates, knowledge-awareness-practice gaps, and healthcare provider influence

**DOI:** 10.3389/fonc.2026.1890017

**Published:** 2026-09-16

**Authors:** Eman Alshehri, Malak AlHakeem, Remaz Abdullah Almahmoud, Aleen Abdulaziz AlKulyah, Ariam Majed Almasari, Aishah Ibrahim Boureggah, Aroub Mohammed Almahmoud, Maryam Ali Alghannam, Aseel Fahad Alsaif, Ahmed Sherif AbdelHamid Abdelwahab

**Affiliations:** 1 Department of Obstetrics and Gynecology, College of Medicine, King Saud University, Riyadh, Saudi Arabia; 2 College of Medicine, King Saud University, Riyadh, Saudi Arabia; 3 Department of Obstetrics and Gynecology, King Saud University, Riyadh, Saudi Arabia

**Keywords:** cancer epidemiology, cervical cancer, HPV vaccination, pap smear, preventive medicine, Saudi Arabia

## Abstract

**Background and purpose:**

Cervical cancer prevention continues to pose a significant public health challenge in Saudi Arabia. This research aimed to evaluate women’s knowledge, awareness, and behaviors related to cervical cancer screening and Human papillomavirus (HPV) vaccination in Riyadh, and to identify key factors influencing participation and the obstacles faced.

**Methods:**

This cross-sectional study was conducted among a diverse sample of women recruited through online platforms, institutional networks, and community-based groups. Data collection involved a structured questionnaire covering sociodemographic details, knowledge, awareness, practices, and barriers. Data analysis included descriptive statistics, gap analysis, and multivariable regression using the Statistical Package for the Social Sciences (SPSS) v28.0.

**Results:**

Awareness of Papanicolaou (Pap) smear was high at 77.8%, but actual screening participation was low at 26.7%. HPV vaccine awareness was 42.0%, yet only 9.3% had received the vaccine. Knowledge levels varied: 82.0% understood the purpose of the Pap smear, but just 20.4% knew the recommended screening intervals. The main barriers included a lack of understanding (43.4%) and limited awareness of testing locations (17.1%). Regression analysis showed that awareness of the HPV vaccine significantly predicted knowledge (β = 1.903, p < 0.001), and that advice from physicians doubled vaccination rates compared with other sources.

**Conclusions:**

A notable gap exists between awareness and practice, mainly due to knowledge deficits rather than access issues. Future efforts should focus on provider-led, behavior-oriented education to convert awareness into action and lessen the cervical cancer burden.

## Introduction

Human papillomavirus (HPV) infection poses a significant challenge worldwide, being the most common sexually transmitted infection and a key cause of various cancers such as cervical, oropharyngeal, and anogenital cancers (1). With an estimated global prevalence of 11.7% among women with normal cervical cytology, most infections are asymptomatic, which means they often go unnoticed and can be transmitted unknowingly. This highlights the vital importance of getting vaccinated before starting sexual activity to ensure the best protection (2, 3). Although effective vaccines have been available for nearly twenty years, many regions, including the Middle East, still see low vaccination rates, creating a gap between vaccine availability and people’s willingness to receive it (4).

Cervical cancer, primarily associated with HPV, continues to be a major public health issue. Globally, it is a leading cause of cancer-related illness among women aged 15 to 44 (5). In Saudi Arabia, there are roughly 350–400 new cases each year, highlighting the importance of effective prevention strategies at both the primary and secondary levels (6). These statistics highlight the need to move from outdated estimates to current, evidence-based monitoring. The situation is further complicated by factors like the shift towards comprehensive nationwide screening and vaccination initiatives, as well as societal and behavioral factors, with HPV prevalence among women reported to range from 4.7% to 30.4% (7, 8).

Saudi Arabia has taken steps to address this issue by including the HPV vaccine in the childhood immunization schedule for girls and setting up screening guidelines (9). Under the guidance of the Saudi Ministry of Health, the national immunization program now includes the Human papillomavirus (HPV) vaccine, which is routinely offered to girls aged 11 to 13 years, with catch-up doses provided based on clinical need. Concurrently, the cervical cancer screening protocol emphasizes opportunistic Papanicolaou (Pap) smear testing for women aged 25 to 65 years, ideally conducted every three to five years, in alignment with international best practices. These national efforts represent a strategic transition toward systematic prevention; however, as noted, their impact remains contingent upon public participation and awareness. However, only about 30% of the targeted population participates in these programs, leaving public health goals unmet (10). This gap persists despite the vaccine’s availability and reflects broader regional challenges, with a significant gap between parents’ willingness to vaccinate their daughters and the actual vaccination rates observed across the Gulf Cooperation Council (GCC) region (4).

Research indicates that low awareness, safety concerns, and cultural barriers significantly hinder vaccine acceptance in Gulf countries (1). A recent systematic review consistently shows that these issues influence parents’ vaccination decisions across GCC countries, while another study found that 77% of women diagnosed with cervical cancer in Saudi Arabia were infected with HPV. Sadly, many of these women only seek treatment at advanced stages due to a lack of knowledge and screening (8, 11). However, many studies focus narrowly on specific groups or combine data from different countries, which can obscure important sociodemographic and knowledge-related factors within the broader Saudi female population.

While existing literature has documented the prevalence of cervical cancer awareness in Saudi Arabia, there is a critical dearth of research investigating why, despite increasing awareness, screening and vaccination rates remain stagnant. This study bridges this gap by shifting the focus toward the behavioral drivers and the hierarchical effectiveness of different information sources in Riyadh.

This study uses the Health Belief Model (HBM) to examine the cognitive and behavioral factors that influence participation (12). The HBM offers a strong framework for understanding how perceptions of susceptibility to cervical cancer, disease severity, benefits of Pap smears and vaccination, and barriers to accessing these services impact health-seeking behaviors. Using this model helps us better understand the disconnect between overall awareness and actual clinical participation in Riyadh.

This study aims to address these critical gaps by conducting a comprehensive assessment of knowledge, awareness, and practices regarding cervical cancer prevention among women in Riyadh.

## Patients and methods

### Study design and setting

This study employed a community-based, cross-sectional analytical design to assess knowledge, awareness, and practices regarding cervical cancer prevention. The study protocol was reviewed and granted ethical approval by the Institutional Review Board (IRB) of the College of Medicine, King Saud University (Reference Number: 26/0170/IRB).

The study was conducted in full accordance with the ethical principles outlined in the Declaration of Helsinki. Prior to participation, all eligible women were provided with a detailed information sheet explaining the study’s purpose, procedures, risks, benefits, and their rights. Electronic informed consent was obtained from every participant prior to the commencement of the survey.

Target Population and Recruitment Strategy: The target population included women aged 18 and above living in Riyadh, Saudi Arabia. We used a convenience sampling method, distributing the survey online via social media platforms such as Twitter, WhatsApp, and LinkedIn, as well as through institutional networks, including students and staff at King Saud University and local community groups. This strategy aimed to reach a broad, urban cohort of women outside hospital settings.

Given the convenience sampling approach using digital platforms (Twitter, WhatsApp, and LinkedIn) and open institutional distribution, the total number of individuals reached is not quantifiable. Consequently, a traditional response rate cannot be calculated. However, our recruitment strategy remained active until the predefined target sample size of 450 completed questionnaires was achieved, ensuring the study was sufficiently powered for the intended statistical analyses.

### Inclusion and exclusion criteria

The inclusion criteria included: (1) women aged 18 years or older; (2) living in Riyadh at the time of the study; and (3) consent to participate electronically. Exclusion criteria comprised: (1) living outside the Riyadh province; and (2) not completing the required sections of the questionnaire.

### Study instrument

The questionnaire was adapted from a validated Arabic instrument created by Zahid et al. (13) for their Madinah study. Due to cultural and linguistic similarities between Madinah and Riyadh populations, the instrument was directly applicable. To ensure it suited our specific goals, we conducted a pilot with 30 women (not included in the final analysis) to check clarity and relevance. The internal consistency of the 20-item knowledge scale was measured with Cronbach’s alpha, which was 0.78, indicating high reliability for our sample.

Building on Zahid et al. (13)’s effective digital dissemination approach, we adopted a community-based convenience sampling method. The survey was shared through digital channels such as WhatsApp, Twitter, and LinkedIn. To capture a wide cross-section of Riyadh’s population, we utilized institutional networks at King Saud University and promoted the survey across different community groups, including university students and residents from various socioeconomic backgrounds within the city.

The questionnaire was structured into distinct sections:

- Section A: Sociodemographic Details gathering information on age, nationality, marital status, educational background, employment, monthly income, and the residential area within Riyadh (see **Table 1**).
- Section B: Awareness and Practices exploring previous knowledge of the Pap smear test and HPV vaccine, personal history of screening and vaccination, perceived obstacles to Pap smear uptake, and sources of information (see **Table 2**).
- Section C: Knowledge Evaluation assessing understanding of cervical cancer risk factors, screening guidelines (including frequency and location), and HPV vaccine information such as effectiveness, recommended age, and cost, through multiple-choice and true/false questions. An overall knowledge score was derived from the total correct answers (see **Table 3**).

**Table 1:** Sociodemographic characteristics of the study participants (N = 450).

Characteristic (or Sociodemographic Characteristic)	Category (or Subcategory)	Frequency (n)	Percentage (%)
Age group	18–25 years	211	46.9
	26–30 years	18	4.0
	>30 years	221	49.1
Marital status	Never Married	211	46.9
	Ever Married	239	53.1
Education level	High School or Less	69	15.3
	University Degree	352	78.2
	Higher Studies	29	6.4
Employment	Housewife	72	16.0
	Student (Non-Biomedical)	58	12.9
	Student (Biomedical)	78	17.3
	Employed (Non-Healthcare)	179	39.8
	Employed (Healthcare)	16	3.6
	Private Work	47	10.4
Monthly income (SAR)	<5,000	22	4.9
	5,000-9,000	54	12.0
	10,000-19,000	144	32.0
	≥20,000	230	51.1

**Table 2:** Awareness, uptake, and perceived barriers to cervical cancer prevention (N = 450).

Measure	Variable	n	%
Awareness	Heard of Pap smear (Yes)	350	77.8
	Heard of HPV vaccine (Yes)	189	42.0
	Believe HPV vaccine is effective (Yes)	189	42.0
	Know vaccine is provided free (Yes)	257	57.1
Uptake	Have done Pap smear (Yes)	120	26.7
	Have taken HPV vaccine (Yes)	42	9.3
Barriers (pap smear)	Do not know why it should be done	147	43.4
	Other reasons	120	35.4
	Do not know where it is available	58	17.1
	Shyness and embarrassment	46	13.6
	Believe it is not effective	9	2.7
Information source	Awareness campaigns	141	31.3
	Social media channels	136	30.2
	No ultimate sources	123	27.3
	Governmental hospital physician	52	11.6
	Private hospital physician	22	4.9
Among those who never had pap smear (n=330)			

**Table 3:** Knowledge assessment of cervical cancer risks, screening, and prevention (N = 450).

Knowledge item	Correct answer	Correctly answered n (%)	Incorrectly answered n (%)
Pap smear & screening			
Pap smear is a screening test for cervical cancer	True	369 (82.0)	81 (18.0)
Screening should be repeated every 3 years	True	92 (20.4)	358 (79.6)
Can be performed at primary healthcare centers	True	350 (77.8)	100 (22.2)
Risk factors			
Family history is a risk factor	True	337 (74.9)	113 (25.1)
Viral infections (e.g., HPV) are a risk factor	True	268 (59.6)	182 (40.4)
Multiple childbirths is a risk factor	True	32 (7.1)	418 (92.9)
Long-term contraceptive use is a risk factor	True	132 (29.3)	318 (70.7)
Smoking is a risk factor	True	189 (42.0)	261 (58.0)
HPV vaccine			
The HPV vaccine is effective at preventing cervical cancer	True	189 (42.0)	261 (58.0)
The vaccine should be administered at age ≤25 years	True	191 (42.4)	259 (57.6)
The vaccine is provided free of charge	True	257 (57.1)	193 (42.9)

### Sample size justification

The required sample size was calculated to ensure adequate precision for estimating prevalence in a cross-sectional design using the formula: n = Z^2 ·^. P(1−P)/d^2^, where Z represents the confidence level (1.96 for 95%), P is the anticipated prevalence, and d is the margin of error. Based on findings by Zihad et al. (13), which reported a Pap smear uptake of 12.5% in a comparable Saudi population, we set P = 0.125 and d = 0.03. This yielded a required sample size of 448 participants. A final sample of 450 participants was recruited, ensuring the study was sufficiently powered to estimate key prevalence metrics within the specified margin of error while allowing for a negligible non-response rate.

### Data analysis

We analyzed the data with IBM SPSS (Statistical Package for the Social Sciences) version 28.0. Descriptive statistics included frequencies and percentages for categorical variables and means and standard deviations (SD) for continuous variables. Knowledge was measured using a 20-item scale (range: 0–20), with 1 point for each correct answer. For comparison purposes, we used the sample mean (5.78 ± 2.16) as a cutoff, assigning participants to the “above-average” group (score > 5.78) or the “average or below-average” group (score ≤ 5.78).

Multivariable linear regression was used to explore predictors of total knowledge scores. To examine factors related to awareness and behaviors regarding Pap smears and HPV vaccines, we applied three separate binary logistic regression models and reported adjusted odds ratios (aORs) with 95% confidence intervals. Additionally, we conducted a gap analysis by calculating the percentage-point differences between awareness and practice. Comparative analyses of information sources and outcomes used one-way Analysis of Variance (ANOVA) and chi-square tests. All tests used a p-value < 0.05 as the threshold for statistical significance, with model assumptions (e.g., multicollinearity, linearity, and homoscedasticity) verified.

To ensure data quality, we set all key items in our digital questionnaire as mandatory responses to prevent partial submissions. Incomplete questionnaires at the time of submission were automatically excluded from the final dataset. As a result, no imputation methods were necessary because only fully completed questionnaires were included in the final analysis.

## Results

The cohort is predominantly Saudi (94%), highly educated (84.6% with a university degree or higher), and with high household income (51.1% earning ≥20,000 Saudi Riyal (SAR)/month). The sample is almost evenly split between younger (18-25) and older (>30) women, and between never-married and ever-married individuals, providing a robust demographic base for analysis across these key strata.

A notable disparity between awareness and practice was observed: while 77.8% are aware of Pap smears, only 26.7% have undergone one. The primary barrier is notable knowledge gaps; 43.4% don’t understand the test’s purpose. HPV vaccine uptake is critically low (9.3%) despite higher awareness of its free provision (57.1%). Traditional and digital awareness campaigns are the main information sources, yet over a quarter of women have no reliable source, highlighting a crucial channel for intervention.

**Figure 1** shows that direct communication with healthcare providers (physicians in both government and private settings) is significantly and positively correlated with higher prevention-related metrics. Cohorts citing physicians as their primary source reported the highest mean knowledge scores and exhibited a substantially higher proportion of Pap smear uptake than those relying on social media or general campaigns.

**Figure 1:**
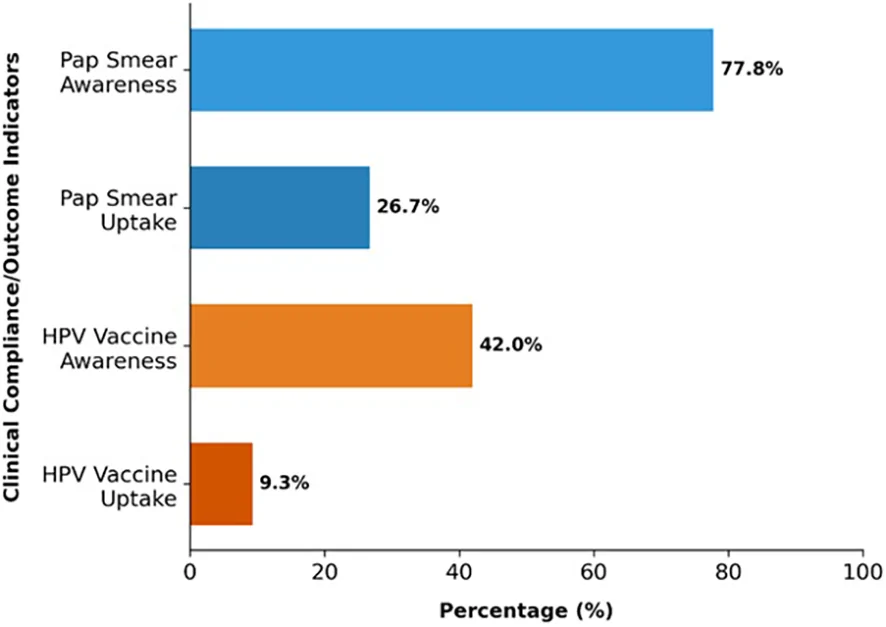
The awareness-practice chasm in cervical cancer prevention.

**Figure 1** quantifies a significant gap between awareness and practice. For Pap smears, a 51.1-percentage-point drop-off occurs between awareness (77.8%, n=350) and uptake (26.7%, n=120). Similarly, a 32.7-point gap exists for the HPV vaccine (42.0% awareness vs. 9.3% uptake). McNemar’s test confirms these declines are significant (p<0.001), showing passive awareness is insufficient and interventions must target specific behavioral barriers.

Knowledge is highly variable and often poor. While most women correctly identify a Pap smear’s purpose (82.0%) and where to get one (77.8%), critical gaps exist: only 20.4% know the recommended 3-year screening interval. Knowledge of risk factors is very low, especially for multiple childbirths (7.1% correct) and long-term contraceptive use (29.3%). Crucially, less than half correctly understand the HPV vaccine’s efficacy (42.0%) or appropriate age for administration (42.4%), identifying key educational priorities for effective prevention.

Descriptive statistics used frequencies and percentages, while the gap analysis measured percentage-point differences. **Table 4** highlights the disconnect between cognitive awareness and behavioral practice. A notable disparity between awareness and practice was observed: while 77.8% were aware of Pap smears, only 26.7% had undergone one. The primary barrier identified was limited comprehension. HPV vaccine uptake was 9.3%, despite 57.1% awareness of its availability for free. Direct communication with healthcare providers was associated with higher mean knowledge scores and higher rates of Pap smear uptake compared to information received via social media. Multivariable regression analyses identified factors associated with specific outcomes. Being over 25 years old and having a family history of cervical cancer were significant predictors of screening uptake. Regarding vaccine uptake, a higher knowledge score was positively correlated with vaccination.

**Table 4:** Knowledge-awareness-practice gaps in cervical cancer prevention (N = 450).

Domain	Metric	n	%	Gap analysis
Awareness	Heard of pap smear	350	77.8	–
	Heard of HPV vaccine	189	42	–
Knowledge	Above-average knowledge (Score > 5.78)	225	50.0	–
	Believe vaccine is effective	189	42	–
Practice	Pap smear uptake	120	26.7	-51.1 pp
	HPV vaccine uptake	42	9.3	-32.7 pp
Barriers	Lack of understanding (purpose)	147	43.4	–
	Lack of access (location)	58	17.1	–
	Psychosocial (shyness/embarrassment)	46	13.6	–

Knowledge categorized based on sample mean (cutoff > 5.78, SD = 2.16); pp = percentage points.

Multivariable linear regression (using unstandardized β coefficients) analyzed the continuous knowledge score, while multivariable binary logistic regression (providing adjusted odds ratios, aOR) examined the binary outcomes of awareness and uptake (**Table 5**). All models were mutually adjusted for baseline sociodemographic covariates. This analysis indicates that different factors influence various stages of knowledge, awareness, and practice. Hearing about the HPV vaccine was the strongest positive predictor of both the overall knowledge score (β= 1.903, 95% CI: 1.505–2.302; p < 0.001) and awareness of Pap smear (aOR = 4.532, 95% CI: 2.400–8.556; p < 0.001).

**Table 5:** Multivariable predictors of key cervical cancer prevention outcomes.

Predictor	Outcome: knowledge score unstandardized β (95% CI); p-value	Outcome: pap smear awareness aOR (95% CI); p-value	Outcome: pap smear uptake aOR (95% CI); p-value	Outcome: HPV vaccine uptake aOR (95% CI); p-value
Demographic factors				
Age group (>25 years)	Not included in model	2.503 (1.797–3.485); <0.001	4.463 (2.929–6.801); <0.001	0.363 (0.194–0.679); 0.002
Ever married	-0.720 (-1.078 to -0.363); <0.001	Not included in model	Not included in model	Not included in model
Awareness & history				
Heard of pap smear (yes vs no)	0.525 (0.087–0.962); 0.019	Dependent Variable	6.131 (1.697–22.157); 0.006	1.160 (0.373–3.609); 0.798
Heard of HPV vaccine (yes vs no)	1.903 (1.505–2.302); <0.001	4.532 (2.400–8.556); <0.001	1.712 (0.791–3.707); 0.172	7.112 (0.840–60.203); 0.072
Family history of CC (yes vs no)	-0.334 (-0.896 to 0.228); 0.243	0.673 (0.231–1.961); 0.468	3.408 (1.449–8.015); 0.005	5.315 (1.854–15.235); 0.002
Behavior & Scores				
Done pap smear test (yes vs no)	-0.083 (-0.284 to 0.117); 0.414	2.141 (1.380–3.321); 0.001	Dependent variable	3.318 (1.005–10.962); 0.049
Taken HPV vaccine (yes vs no)	1.319 (0.955–1.684); <0.001	0.918 (0.502–1.677); 0.781	1.504 (0.686–3.300); 0.308	Dependent variable
Overall knowledge score	Dependent variable	1.188 (1.015–1.390); 0.032	0.952 (0.809–1.121); 0.556	1.592 (1.270–1.996); <0.001

Behavioral practice (uptake) is influenced by different factors: older age (aOR = 4.463, 95% CI: 2.929–6.801; p < 0.001) and a family history of cervical cancer (aOR = 3.408, 95% CI: 1.449–8.015; p = 0.005) strongly predicted Pap smear screening. In contrast, HPV vaccine uptake was mainly associated with higher baseline knowledge (aOR = 1.592, 95% CI: 1.270–1.996; p < 0.001) and a positive family history of cervical cancer (aOR = 5.315, 95% CI: 1.854–15.235; p = 0.002). Simply knowing about a Pap smear does not ensure its clinical use, underscoring that public health strategies should be tailored to distinct behavioral goals rather than relying solely on awareness campaigns.

Univariate analyses showed significant differences between information channels. A one-way ANOVA indicated that average knowledge scores varied significantly by source (F(4, 445) = 14.83, p < 0.001). *Post-hoc* Tukey HSD tests confirmed that groups informed by physicians scored higher than those relying on social media or having no reliable source. Likewise, Pearson’s Chi-square tests of independence found significant connections between the information source and actual health actions (X 2(4) = 21.41, p < 0.001 for Pap smear uptake; X 2(4) = 9.68, p = 0.046 for HPV vaccine uptake). These univariate results highlight a statistically significant association between the reported information source and preventive health behaviors. However, it’s important to note that mass media and social media remain the predominant channels reaching the population (**Table 6**).

**Table 6:** Primary information sources and their association with knowledge and practice.

Information source	Users n (%)	Mean knowledge score (SD)	Pap smear uptake (%)	HPV vaccine uptake (%)
Awareness campaigns	141 (31.3)	6.1 (2.0)	32.6	11.3
Social media	136 (30.2)	5.5 (2.3)	22.1	7.4
No reliable source	123 (27.3)	4.9 (2.1)	18.7	5.7
Governmental physician	52 (11.6)	7.2 (1.8)	48.1	15.4
Private physician	22 (4.9)	7.0 (1.9)	45.5	13.6
Statistical significance	N/A	p < 0.001 a	p < 0.001 b	p = 0.046 b

*a* Calculated using a One-way Analysis of Variance (ANOVA) to compare mean knowledge scores (F = 14.83).

*b* Calculated using Pearson’s Chi-square (X^2^) test of independence to compare behavioral uptake proportions (X^2^ = 21.41 for Pap smear; X^2^ = 9.68 for HPV vaccine).

**Figure 2** illustrates the descriptive relationship between information channels, knowledge scores, and screening practices. While raw knowledge scores and screening rates peak among physician-informed cohorts, mass media and organized campaigns retain the greatest overall reach across the population. Rather than indicating an absolute hierarchy, these channels act in complementary ways: broad media platforms drive widespread baseline public exposure, whereas direct engagement with healthcare providers is strongly associated with higher rates of completed screening.

**Figure 2:**
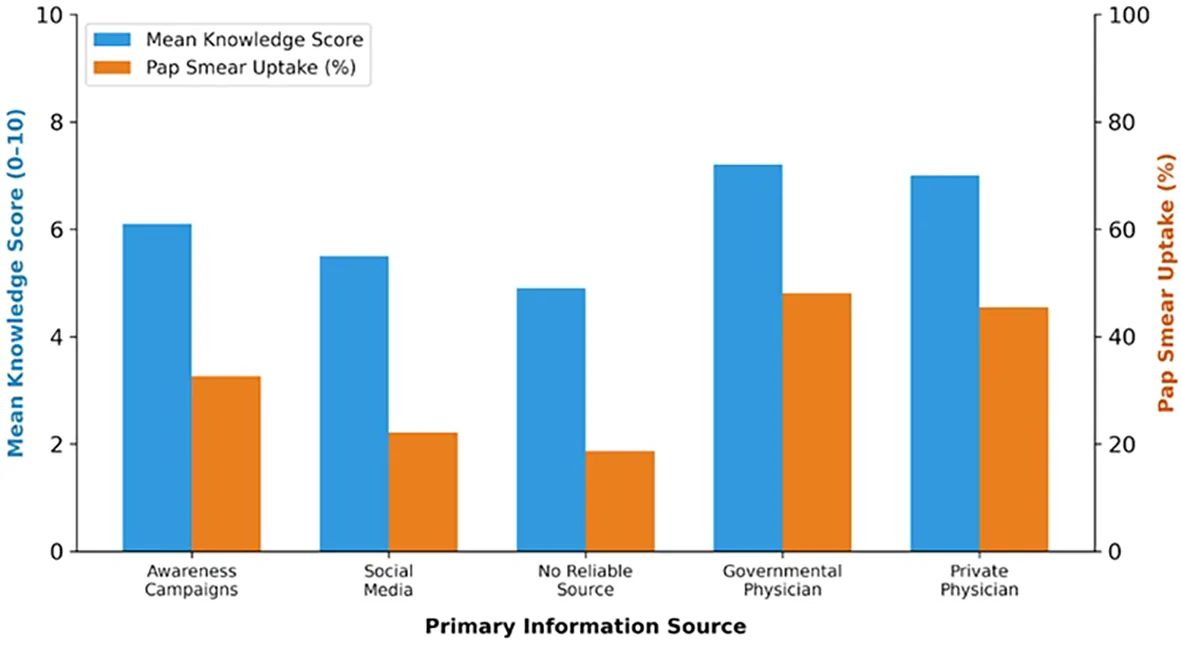
Influence of information sources on knowledge and practice.

## Discussion

### Our results and their interpretation

The study uncovered a significant gap between awareness and actual preventive action. While 77.8% of women (n=350) were aware of Pap smear screening, only 26.7% (n=120) had undergone the procedure, highlighting a stark 51.1-percentage-point gap. Awareness of the HPV vaccine was present in 42.0% (n=189) of participants, yet only 9.3% (n=42) had received it, resulting in a disparity of 32.7 percentage points. This large gap suggests that although health messages have reached the population, they are not yet associated with widespread engagement in preventive care, posing a major public health concern.

While previous regional studies have established baseline cervical cancer awareness in Saudi Arabia, this study examines factors contributing to the ‘awareness-practice chasm,’ revealing a 51.1% gap in Pap smear uptake and a 32.7% gap in HPV vaccination. Findings suggest that public health strategies face a ‘behavioral ceiling,’ associated with a deficiency in understanding of purpose (43.4%) and reliance on suboptimal information channels. Multivariable modeling shows that reporting physician-led counseling as a primary information source is more strongly associated with screening uptake than reliance on digital campaigns, supporting a policy interest in exploring provider-patient dynamics. This provides clinicians with data highlighting which information channels track most closely with actual preventive practices.

Granular exploration of participant knowledge revealed crucial nuances behind the baseline numbers. While 82.0% of participants correctly identified the purpose of a Pap smear, only 20.4% knew the recommended three-year screening interval. Similarly, 42.0% recognized the efficacy of the HPV vaccine, but there were gaps in understanding of vaccination timelines. These findings highlight that superficial public exposure does not ensure functional health literacy. This underscores the need for structured education that goes beyond basic awareness to address scheduling and clinical benefits.

The variability in knowledge about cervical cancer risk factors has significant clinical and public health implications. While most participants recognized family history (74.9%) and HPV (59.6%) as risks, many were unaware of reproductive and behavioral factors. Notably, 92.9% did not see multiple childbirths as a risk, and 70.7% were unaware that long-term oral contraceptive use increases risk. This highlights a critical gap in regional health literacy. In clinical settings, prolonged oral contraceptive use and high parity are known to promote cervical cancer by working with persistent high-risk HPV infections, accelerating carcinogenesis through hormonal pathways that boost viral gene expression. When women are unaware of these links, they tend to underestimate their risk, especially if they have many children or use contraception long-term but lack a cancer family history.

Furthermore, the fact that 58.0% of participants did not recognize smoking as a risk factor highlights a significant gap in preventative awareness. Tobacco byproducts concentrate in cervical mucus, causing localized DNA damage and impairing cell-mediated immunity, which halves the clearance rate of high-risk HPV strains. These findings suggest that public health efforts should shift from vague cancer warnings to explicit, mechanistic education about behavioral and reproductive risk factors. Primary care physicians should utilize routine family planning and smoking cessation visits to screen high-risk women unaware of their vulnerability.

The multivariable regression models identified key factors influencing behavioral outcomes. Prior awareness of the HPV vaccine was the strongest positive predictor of overall knowledge scores (β = 1.903, p < 0.001). For screening practices, age over 25 (aOR = 4.463, p < 0.001) and a family history of cervical cancer (aOR = 3.408, p = 0.005) were significant predictors of Pap smear uptake. HPV vaccine uptake was mainly driven by higher objective knowledge (aOR = 1.592, p < 0.001), family history (aOR = 5.315, p = 0.002), and previous screening experience (aOR = 3.318, p = 0.049). Older age was a negative predictor for vaccine uptake (aOR = 0.363, p = 0.002), suggesting younger groups are more receptive to immunization.

A key paradox in our multivariable linear regression is that being “ever married” negatively predicts cervical cancer knowledge scores (β = -0.720, p < 0.001). This trend reflects a generational and policy shift in Saudi Arabia, where public health campaigns and HPV vaccine awareness efforts over the past decade mainly targeted younger, single women, particularly those aged 18–25, who make up 46.9% of our sample. As a result, younger, unmarried women have had more digitally driven exposure to reproductive health prevention.

In Saudi Arabia, married women often approach reproductive care reactively rather than preventively, especially those who married before recent health media campaigns. Clinical visits typically focus on pregnancy, antenatal care, and immediate fertility issues. Preventative topics like cancer screening or STI transmission are rarely discussed unless symptoms appear. Cultural modesty and taboos surrounding STIs, such as HPV, can prevent women and providers from discussing or seeking preventative information. Despite frequent contact with gynecological services, marriage can act as a barrier to accessing comprehensive health literacy.

An analysis of information channels highlighted differences in clinical outcomes across subgroups. Those relying on government or private physicians had higher knowledge levels and screening rates (48.1% and 45.5%) than social media users (22.1%) or those with no reliable sources (18.7%). However, these results should be interpreted carefully. The subgroups citing physicians as their main source were relatively small (11.6% for government and 4.9% for private physicians) versus 30.2% for digital media users. While these notable univariate findings indicate that direct interactions with providers are strongly linked with positive health behaviors, the small sample sizes limit the extent to which these findings can be generalized across the entire population. Instead of viewing these channels as mutually exclusive, our data suggest they coexist: digital platforms provide broad exposure, while reporting provider interactions as a source co-occurs with higher clinical compliance.

Identifying explicit barriers among non-screeners offers a clear path for public health strategy. The main obstacle was a knowledge gap: 43.4% (n=147) of non-screeners lacked an understanding of the test’s clinical importance. Logistical issues, such as unfamiliarity with facilities (17.1%, n=58), and psychosocial factors like shyness or cultural embarrassment (13.6%, n=46), also hindered screening. Addressing these barriers requires a combined approach: effective education on screening benefits, structural improvements to accessibility, and private, supportive environments to reduce patient hesitancy.

### Comparison of our results to similar studies

Our data indicate a measurable difference between awareness (77.8%) and clinical practice (26.7% Pap smear uptake), a pattern also observed in regional literature. Alrajeh & Alshammari (14) reported a more pronounced variation in Riyadh primary care clinics, where vaccine awareness was lower (21.5%) and uptake was 3.4%. This trend suggests that despite potential improvements in awareness initiatives over time, the challenge of translating knowledge into consistent clinical action remains a significant public health consideration among Riyadh’s female population across various clinical settings.

The precise knowledge deficits we identified, particularly regarding vaccination specifics (42% knew efficacy/age), are a consistent theme. Gari et al. (15) in Makkah found similarly low levels of detailed knowledge among students, with only 16.5% aware of the optimal age for vaccination. However, our cohort demonstrated a stronger baseline awareness of the Pap smear’s purpose (82%) than Gari’s study, where only 44.9% had heard of it. This difference may be attributed to our sample’s older age and higher proportion of ever-married women, a demographic profile often associated with greater exposure to gynecological healthcare information.

When viewed within a national context, our findings are consistent with the larger Saudi landscape. Barhamain and Alwafi’s (16) nationwide research revealed nearly identical levels of limited HPV knowledge, 54.8%, compared to roughly 50% in our study, where scores were 5.78 or lower, and a low rate of vaccine uptake, at 3.0% versus our 9.3%. The somewhat higher vaccination rate we observed might be linked to regional differences or the fact that our data collection occurred later, capturing some of the early benefits of increased national efforts to promote HPV awareness. Nevertheless, vaccination levels remain well below public health goals.

Bakhashab et al. (17) and Alshrari (18) further support that these gaps persist, pointing out that although general awareness of HPV grows, understanding of how the disease is transmitted and the vaccine schedule remains inadequate. Their studies indicate that “awareness” tends to be superficial and does not lead to the behavioral changes necessary for completing the vaccine series. Similarly, Alshahrani et al. observed that parents often misinterpret the vaccine’s purpose, with many thinking it is unnecessary if their children are not yet sexually active. This highlights the urgent need for more targeted, age-appropriate educational programs, consistent with our findings.

A common theme across these studies is the hierarchy of sources influencing health behaviors. Our results strongly indicate that physician guidance is a significant factor associated with higher vaccine uptake, with rates of 45–48%. This aligns with findings from Turki and Alqurashi (19), who also emphasized healthcare professionals as trusted and preferred sources of information. Both studies demonstrate that social media and general awareness campaigns show weaker associations with behavioral execution. The consistency of these statistical patterns across different regions, Riyadh and Makkah, suggests that relying solely on broad awareness campaigns may yield different outcomes than approaches integrated with professional medical guidance, though interventional studies are needed to confirm a directional effect.

This professional-led approach is supported by Bakhsh et al. (20), who observed that medical students with direct access to clinical education were significantly more likely to pursue vaccination. Furthermore, Alherz et al. and Algaadi et al. reinforce this by highlighting that individuals who rely on healthcare teams for information exhibit higher levels of vaccine confidence than those who primarily use social media. This reinforces the notion that the “trusted intermediary”—the clinician—is the most effective vehicle for overcoming hesitancy and ensuring that patients receive accurate, actionable information.

Lastly, our conclusion that misunderstanding is the key barrier is well-supported. We found that 43.4% of participants did not know the purpose of screening. Similarly, Azzi et al. (21) conducted a large nationwide survey, which found that 57.9% of women cited a lack of information as their main obstacle. The recurring nature of this finding across multiple studies, diverse sample sizes, and various time periods points to a systemic gap in the delivery of health education. This indicates that simply repeating awareness messages is insufficient; instead, it is crucial to fundamentally redesign educational approaches to better communicate the importance, purpose, and process of cervical cancer prevention in a way that resonates with the community.

To address these gaps, we propose three tailored interventions in Saudi healthcare. First, an automated cervical screening module should be added to the Sehhaty app, offering personalized infographics and convenient booking at local clinics. Second, primary care should incorporate prompts in Electronic Health Records to ensure brief, provider-led preventive counseling during family planning or antenatal visits. Lastly, female-led “Clinic-in-a-Box” pop-ups in public spaces can provide private, peer-to-peer education, transforming digital awareness into practical health actions.

### Strengths and limitations

One notable strength of this study is its large sample size of 450 participants, which surpasses many similar regional investigations. This robust number allowed for detailed multivariable analyses, such as linear and logistic regression, to identify key factors correlated with knowledge and uptake. The study’s focus on factors contributing to the knowledge-awareness-practice gap, specifically highlighting the hierarchical influence of information sources, provides insights for potential consideration that pivot from passive awareness-raising to evidence-based, provider-led clinical recommendations.

On the other hand, the study has some limitations. Its cross-sectional design means it cannot determine cause-and-effect relationships between variables. This study’s results should be viewed with caution due to the participant demographic, which mainly consisted of highly educated Saudi nationals from higher socioeconomic backgrounds. Our digital convenience sampling introduced selection bias that limits the generalizability of these findings to the broader female population in Riyadh and Saudi Arabia, particularly among those with lower education levels, lower household incomes, or limited digital access. Therefore, while our research offers valuable insights into an urban, educated group, it likely underrepresents the challenges faced by more vulnerable or marginalized populations, who may encounter even greater systemic barriers to cervical cancer prevention. Also, while the study assessed knowledge levels, it may not fully capture a deep understanding of complex topics through a quantitative questionnaire alone.

Looking ahead, future research should include a longitudinal cohort study to explore how knowledge correlates with screening and vaccination behaviors over time, thereby facilitating a more robust analysis of trends. Incorporating qualitative methods, such as interviews or focus groups, can reveal underlying psychological, cultural, and structural barriers. Investigating targeted educational programs led by healthcare providers is also essential to address trends in vaccine and screening uptake. Moreover, nationwide surveys using stratified random sampling are needed to validate and extend these findings across Saudi Arabia’s diverse populations.

In conclusion, this study highlights a significant gap: high awareness does not necessarily translate into high uptake of cervical cancer prevention measures in Riyadh. Key obstacles include knowledge gaps and misconceptions, beyond mere access issues. Future efforts may benefit from shifting focus toward awareness campaigns and focus on provider-led, behavior-oriented education strategies to effectively bridge this gap.

## Data Availability

The raw data supporting the conclusions of this article will be made available by the authors, without undue reservation.
